# Supplementary material for: Screening of Cognitive Changes in Adults with Intellectual Disabilities: A Systematic Review
Source: Brain Sci. 2020 Nov 12;10(11):848. doi: 10.3390/brainsci10110848 (PMC7698112; doi:10.3390/brainsci10110848)
Supplement: Supplementary file 1 [file brainsci-10-00848-s001.zip › brainsci-964520-Sup/Cross sectional studies_CT.docx]

**Table A2**. Cross-Sectional Studies assessing cognitive changes in ID and ID-DS participants

| Article | Study city/country | Population | Instruments | Comparison | Outcomes | Quality Assessment Scale  (0/8) |
| --- | --- | --- | --- | --- | --- | --- |
| Devinsky, Sato, Conwit, Schapiro (1990) | USA | DS n=28  N=19 young adults  N=9 older adults | PPVT-R, Block Pattern subtest of HNTLA; WISC-R; BDDE; DSF; OPS; BTS; DS | The relation of EEG alpha background to cognitive function | Older patients with DS with decreased alpha waves backgrounds had fewer visuospatial skills, decreased attention span, and dementia | 4 |
| Das & Mishra (1995) | Canada | N=31 DS N=41 NDS | Tasks: VS; MN; RA; NF; SAE; CO; WR; FM; Matrices | Differences between groups | People with DS performed poorly in two verbal tasks; Phonological tasks are more likely to be sensitive in the detection of cognitive decline among people with DS | 5 |
| Das, Divis, Parrila & Naglieri (1995) | USA | DS young: N=16  DS old: N=16  NDS young: N=16  NDS old: N=15 | WAIS or WAIS-R; Stanford-Binet ratio IQ; DRS;  PPVT-R- Form M; MAT; CAS | Examine what intellectual changes between DS and NDS | DS old group performed poorly in most test. In the tasks that involved verbal output both DS groups performed poorly | 5 |
| Nelson et al. (1995) | USA | DS: N=30  DAT: N=18  Elderly controls N=25 | MMSE; NBAP; | Comparison between groups on emotional factors | DS individuals showed more signs of indifference; inappropriateness; pragnosia and scores were consistent also in individuals with DAT | 7 |
| Witts & Elders (1998) | UK | N=33 DS | SIB;  VABS; | The utility of using SIB in the population with DS | For DS and DAT, SIB could be of used in the longitudinal study by comparing age-matched DS and non-DS groups | 4 |
| Crayton, Oliver, Holland, Bradburry & Hall (1998) | UK | N=70 DS; n=39 female, n=31 male. | BPVS; VABS; CAMDEX;ECT | Comparing age groups | Participants with highest risk of developing dementia scored significantly higher in identification test; | 4 |
| Cosgrave, McCarron, Anderson, Tyrrell, Gill & Lawlor (1998) | Ireland | Moderate ID group:  ID/dementia n=19  ID/no dementia n=29  Severe ID group:  ID/dementia n=11 ID/no dementia n=11 | DSMSE  TSI | Comparing scores between groups | The TSI is useful to monitor the progression of dementia longitudinally in severe MR. TSI-Reliability 0.89. | 5 |
| Hon, Huppert, Holland & Watson (1999) | UK | N=77 DS  Group 1 age 30-44 (N=45)  Group 2 age 45 years and over (N=29) | CAMCOG; MMSE | Differences in scores between instruments | Younger group scored higher in the total CAMCOG and MMSE scores on all subtests except Attention/Calculation; CAMCOG can be used when possible dementia is being considered. | 4 |
| Deb & Braganza (1999) | UK | Ntotal=62 DS  N=26 DS/dementia  N=36 DS no dementia | MMSE;  DMR;  DSDS; | Differences in scores between instruments | Positive correlation in the diagnosis of dementia between DMR and the DSDS with specificity and sensitivity at 0.92 in both cases. | 4 |
| Burt, Hart, Phillips, Greene, Loveland, Cummings, Lewis, Lesser, Cleveland & Chen (1999) | UK | N=63 DS;  N=74 NDS;  Mild ID n=27; Moderate ID n=69; Severe ID n=38; profound ID n=4 | DQ;  IBR-MSE | Dementia Questionnaire vs. IBR Mental status exam | Good agreement between DQ and the IBR Mental Status Exam; Disagreement is greater for individuals who are lower functioning and for those with DS; | 5 |
| Devenny, Krinsky-McHale, Sersen & Silverman (2000) | USA | ID/NDS N=40  ID/DS:Healthy N=44  Questionable DAT N=10  Early-Stage DAT N=5  Middle-Stage DAT N=7 | WISC-R;  CRT;  SRT; | Comparison of the amount of decline in different stages of dementia | Group differences: (i.e. healthy with DS, ‘questionable’, early-stage dementia and middle-stage dementia) for each subtest; | 4 |
| Numminen, Service, Ahonen & Ruoppila (2001) | Finland | DS: N=15 group  ID/NDS: N=15 | Taks: DSB; CS; NWR; NWS; DSF; CB; VST; | Compare working memory performance | The DS group performed significantly more poorly in working memory tasks that measured phonological loop; | 5 |
| McCarron, Lawlor & Beagly (2002) | Ireland | No DAT/DS N=14 DS/DAT N=16 | CAS-ID;  DSMSE;  TSI;  DLSQ | Compare the results of the CAS-ID to other validated tests | Good measure cognitive and functional decline in individuals with DS and AD. | 5 |
| Margallo-Lana et al., 2003 | UK | DS N=14 | The Prudhoe Cognitive Function Test (PCFT) | Test inter-rater reliability and to assess test retest reliability; | 0.99 (p<0.01) represents excellent inter-rater reliability to detect cognitive deterioration aspects of dementia. High reliability and temporal stability. | 4 |
| Shultz, et al., 2004 | USA | ID N=38  ID/DS N=26  ID with dementia N=19  ID no dementia N=19 | DSDS;  DMR;  Reiss Screen  Shultz MMSE | Groups with and without diagnosed dementia | Both DSDS and DQMR assess similar elements of dementia; Both subscales of DSDS differentiated between groups; | 5 |
| Nelson, Johnson, Freedman, Lott, Groot, Chang & Head (2005) | USA | DS N=20 | WAIS-III; DMR;  WGTA;  Tasks: ODL, RL; DNMP; DNMS | Differences in scores between instruments | DMR is the strongest predictor of reversal learning error scores, suggesting symptoms of dementia effect on reversal learning; | 4 |
| Burt, Primeaux-Hart, Loveland, Cleveland, Lewis, Lesser & Peterson (2005) | USA | DS N=55;  NDS N=75; | BVSR;  PPVT;  SICD-AASH; LIPS; BSID; TSI-M; PP; DTVMI; DSADS  DSDS; DMR; SoIB; RSMB; PIMRA; DASH-II; DSI; MAS; | DS vs ID no DS individuals; and differences between age groups | Performance of older adults did not change over time, but that of younger adults with DS and adults without DS improved; Adults with DS showed significant and unique declines only in test of verbal fluency; | 5 |
| Beacher et al., (2005) | UK | DS N=48  Control group NDS N=42 | CAMCOG;  BPVS | Comparison between groups | Significant negative correlation between mean myo-inositol concentration and overall cognitive ability in DS group; | 5 |
| Palmer (2006) | USA | Dementia N=10  DS=6; ID/NDS=4  No dementia N=12  DS=4; ID/NDS=8 | CTT; BNT;  TCOWAT; FOME; ESDCL | Comparison between groups | Deficits in the Dementia group in areas consistent with diagnosis of dementia for persons with ID; | 7 |
| Kirk, Hick & Laraway (2006) | UK | ID/NDS N=76  DS N=12 | DMR; ABS | Differences in scores between instruments | DMR gives a general indicator of cognitive and affective symptoms that could indicate dementia. | 5 |
| Pyo, Kripakaran, Curtis, Curtis & Markwell (2007) | USA | DAT N=13  DAT/DS N=6  Normal controls N=31 | OMT; TSI; ABMT;  O; PRT; NEPSY; PPV-III | Comparison between groups | The functional level of the DAT group was significantly lower than that of the normal control group; DAT groups scores significantly lower than the normal group; | 7 |
| Kittler, Krinsky-McHale & Devenny (2008) | USA | DS n=53  Williams syndrome n=10  Mixed etiology n=39 | Short term memory and dual task processing tasks | Comparison between groups | Dual task performance declined significantly in DS; No etiology group differences on single tasks. | 6 |
| Iacono, Torr and Wong (2010) | Australia | N=55 total;  DS N=47; AD or suspected: N=10 | ABDQ; RCPM; PPV; ASM; VSM; TACL-III | Comparison between groups | Adults with DS may show failure in continuing developing in productive syntax. | 5 |
| Ball, Holland, Watson & Huppert (2010) | UK | DS N=78; Mild ID N=33 | CAMCOG; CaD; SR; ToL; SB; CaODB | Changes in behavior | Disinhibited behavior and apathy were both associated with impaired performance in executive function. | 5 |
| Pyo et al., 2010 | USA | DS N=9  NDS N=24  DAT/ DS N=15  DAT/NDS N=11 | r- PRMT; OMT; TSI; NEPSY; | Compared scores between groups | The r-PRMT discriminates between those with DAT rom those without DAT; Controls with DS showed higher scores. | 5 |
| Hutchinson  & Oakes (2011) | UK | DS N=90 (mean age 38.97, SD±9.18)  N=37 males;  N=16 females; | SIB; DMR; | SIB vs DMR criterion validity | The SIB has good concurrent criterion validity when compared to DMR; The SIB has a good validity specifically as a measure of cognitive ability in people with DS because it correlates only with the cognitive functioning component of the DMR. | 4 |
| [De Vreese](https://www.cambridge.org/core/search?filters%5BauthorTerms%5D=Luc%20P.%20De%20Vreese&eventCode=SE-AU) et al., 2011 | Italy | ID N=63  ID/DAT N=15  DAT/DS N=13 | Italian translation of the AADS scale (AADS-I)  DMR; |  | Subjects with DAT scored significantly higher on both DMR subscales compared to the subgroup without DAT; | 4 |
| Head et al. (2011) | USA | DS/NO AD N=17  Normal controls N=11  DS/AD N=17  Normal AD controls N=12  Group 2  DS/AD N=52  DS/NO AD N=78 | BPT (Brief Praxis Test  SIB; DMR | Comparing between groups A beta levels in plasma | No association between plasma Aβ and scores on the SIB and DMR; Lack of sensitivity of SIB and DMR to detect dementia or cognitive decline in DS adults. | 7 |
| Esteba- Castillo et al., 2013 | Spain | ID N=146;  Mild ID N=62;  Moderate ID N=84;  ID/DS N=103  ID/NDS N=43; | DMR; K-BIT I  CAMDEX-DS; CAMCOG-DS | Comparison between instruments | High degree of diagnostic validity between the CAMDEX-DS and the CAMCOG- DS; Reliability scored 0.93. | 4 |
| Knegt et al. (2013) | The Netherlands | DS N=106 (mean age=37; n=56 males)  DS/possible dementia N=49 (age range=40 years and over) | SRZ/SRZ-P ; DMR ; FAS ; CAS ; NRS | Comparison between instruments | Adults with DS have generally a better comprehension of faces rather than numbers and more comprehension of pain affect rather than pain intensity. | 4 |
| Hartley et al. (2014) | USA | DS N=63, n=31 male; n=32 female age range (30-53) | Several neuropsychological batteries | Scores in instruments | No significant differences in measures between DS who had elevated neocortical Pittsburg compound B retention levels and DS who did not. | 4 |
| Koran et al., 2014 | USA | DS N=14  Typically, Development N=82  WS n=41 | DLD  KBIT | Comparing groups | Individuals with DS demonstrated age-related effects on gray matter associated with dementia; | 5 |
| Powell et al., 2014 | USA | NDS N=10  DS no dementia N=10  DS/dementia N=10 | BPT; SIB; | Comparing groups | The BPT test is sensitive to functional declines because of dementia in DS. | 5 |
| De Vreese et al., 2015 | Italy | DS N=36;  NDS N=39 | AFAST; IADL; DMR-I | Comparing groups | AFAST-I fulfils the necessary requirements to become a tool of choice for the evaluation of the basic tasks in adults/seniors with ID and (suspected) dementia. | 4 |
| De Vreese et al. (2015) | Italy | ID N=61  ID/DS N= 22  ID/NDS N=39 | AFAST;  ADL; IADL;  DMR | Pilot analysis of the clinical significance of the AFAST-I | Good internal consistency of the AFAST-I (.92); AFAST-I assesses several difficulty levels of autonomy. | 4 |
| Makary, Testa,Tonge, Einfeld ,Mohr & Gray (2015) | Australia | DS=33 | PPVT-4; DBC-A  ABDQ; ABAS-II |  | Age is associated with decrease in adaptive behavior independent of dementia and health status; Age-related changes are domain specific rather than pervasive; | 4 |
| Walker, MacBryer, Jones & Law (2015) | Netherlands | DS N=26  N=14 More able group  N=12 Less able group | DLD ; DRS-2; VABD-II; ABAS-II; PAS-ADD | Inter-informant agreement | Differences in scores are merely attributable to differing informants’ perspectives. | 4 |
| Walsh et al., 2015 | USA | N=63 men;  mild ID N=40; moderate ID N=44; severe and profound ID N=30  ID/DAT N=71; ID/no DAT N=43 | RADD; DMR; BADLS; SIB; BPT; | Differences between groups | RADD has efficacy for assessing cognitive functions relevant to AD in DS; RADD differentiated participants based on their dementia status; | 5 |
| Benejam et al., 2015 | Spain | DS/no DAT N=75;  DS/DAT N=15 | Modified Cued Recall Test (mCRT) | Memory profiles between groups | Healthy DS achieved higher total scores and commit fewer intrusion errors; In DS- DAT with advanced DAT the mCRT is not useful. | 5 |
| Sinai, Hassiotis, Rantell & Strydom (2016) | UK | DS Total N=49  DS/dementia N=19  DS/no dementia N=30 | ACTB; CANTAB; NAID; ToL; VF; F-NT; GA; OM | Validity of ACTB between groups | Only 3 tests of the ACTB differentiated between demented and non-demented DS groups. | 5 |
| Startin et al., 2016 | UK | Total DS N=128  DS/Dementia N=23/128 | CSDS; CAMDEX; KBIT-2 | Development of CS-DS | Good reliability (0.84) and validity using two raters and over two time points. | 5 |
| de Knegt et al., 2016 | Netherlands | DS N=39 | WPPSI-R; FANT; NPEMID; DMR; SRZ/SRZ-P; FP/FS | Pictogram scales and drawn faces (FAS) | 56% of participants preferred facial pictograms over FAS stimuli; | 4 |
| Lifshitz-Vahav, Shnitzer & Mashal (2016) | Israel | NSD N=18;  DS N=14; | LLPI; PPVS; RSPM; PFT; S; CVMT; IC; TFB; HMGT; MTT; NVMT; TMT | Crystallized and fluid test scores between groups | Participation in cognitively stimulating activities influence cognitive performance in adults with ID with and without DS. | 5 |
| del Hoyo et al., 2016 | Spain | ID N=69  ID/DS N=65/69  COMTVal158Met  N=93; VNTR-DAT1  N=57 | K-BIT; CANTAB; WAIS-III; SFWGT; WCFST; TOLDx | Comparing scores between groups | Met allele carriers showed worse adaptive social skills and self-direction. | 6 |
| Startin et al., 2016 | UK | 36 > age DS/ no dementia  N=130; 36 > age DS/dementia  N=51;16-35 DS N=124 | KBIT-2; Short ABS; DLD; CANTAB; CAMCOG; NAID; ACTB; OMQ; ToL; BRIEF-A; NEPSY; | Scores between groups and instruments | Poor performance for adults with cognitive decline and dementia; Majority of tasks have high completion rate for adults who do not have a diagnosis of dementia; | 4 |
| de Knegt et al., 2016 | Netherlands | DS N=224 | PS; DFPA; WPPSI-R; FANT; NPEMID; FSID; | Pain experience | Structural differences and atypical patterns of brain activation in DS individuals. | 4 |
| García-Alba et al., 2017 | Spain | DS N=63 adults  ID_mild_ N=39 ID_mod N_=24 | KBIT-2; ABS-RC:2; CAMDEX-DS; BT-ID; WCFST; BRIEF; TOLdxrm | Comparing groups | Psychometric properties of the TOL^DXtm^ version for people with ID were satisfactory on all variables; Sensitivity (0.76), Specificity (0.81). | 4 |
| Gomiero et al., 2017 | Italy | N=58 DS; n=40 no dementia; n3=dementia  N=142 NDS, n=126 no dementia; n=2 dementia | DSQIID; DMR | Scores between instruments | Reliability of the DSQIID-I was 0.94; | 5 |
| Majerus & Barisnikov (2018) | Switzerland & Belgium | DS N=47 | EVIP; PN; ISADYLE; STMT; CBTT; NEPSY; RPCM | Vocabulary knowledge verbal abilities | Dissociation between productive and receptive vocabulary measures in verbal short-term memory abilities in DS participants. | 4 |
