# Supplementary material for: Screening of Cognitive Changes in Adults with Intellectual Disabilities: A Systematic Review
Source: Brain Sci. 2020 Nov 12;10(11):848. doi: 10.3390/brainsci10110848 (PMC7698112; doi:10.3390/brainsci10110848)
Supplement: Supplementary file 1 [file brainsci-10-00848-s001.zip › brainsci-964520-Sup/Instruments supplementary Material.docx]

*Table 4. List of Scales, Questionnaires and Inventories*

| Scales, Questionnaires and Inventories |
| --- |
| 1. Adaptive Behaviour Scale–Residential and Community (ABS) 2. Adaptive Behaviour Assessment System-II Adult (ABAS-II) 3. Adaptive Behaviour Dementia Questionnaire (ABDQ) 4. Alzheimer’s Functional Assessment Tool scale for informants (AFAST) 5. Association on Mental Disability Adaptive Behaviour (AMDAB) 6. Bayley Scales of Infant Development (BSID) 7. Behaviour Rating Inventory of Executive Function (BRIEF) 8. Bristol Activities of Daily Living Scale (BADLS) 9. British Picture Vocabulary Scale (BPVS) 10. Caregiver Activity Survey modified (CAS-ID) 11. Cognitive Scale for down Syndrome (CSDS) 12. Daily Living Skills Questionnaire (DLSQ) 13. Dementia Questionnaire (DQ) 14. Dementia Questionnaire for People with Learning Disabilities (DLD/DMR) 15. Dementia Rating Scale (DRS) 16. Dementia scale for Down Syndrome (DSDS) 17. Dementia Screening Questionnaire for Individuals with Intellectual Disabilities (DSQIDD) 18. Developmental Behaviour Checklist – Adult (DBC-A) 19. Diagnostic Assessment for the Severely Handicapped II- DASH II 20. Down Syndrome Mental State Examination (DSMSE) 21. Dyspraxia Scale for Adults with Down Syndrome (DSADS) 22. Early Signs of Dementia Checklist (ESDCL) 23. Facial Pictograms and Facial Scales (FP/FS) 24. Functioning Scale for Intellectual Disability (FSID) 25. Hampshire Social Services Assessment (HSSA) 26. Instrumental Activities of Daily Living (IADL) 27. Italian translation of the AADS scale (AADS-I) 28. Later Life Planning Inventory (LLPI) 29. Leiter International Performance Scale (LIPS) 30. Middlesex Elderly Assessment of Mental State (MEAMS) 31. PAS-ADD Checklist 32. Rapid Assessment for Developmental Disabilities (RAAD) 33. Reiss Screen for Maladaptive Behaviour (RSMB) 34. Scales of Independent Behaviour (SoIB) 35. Sequences Inventory of Communication Development for Adolescents and Adults with Severe Handicaps (SICD-AASH) 36. Shultz Mini Mental State Exam (S-MMSE) 37. Social Functioning Scale for Intellectual Disability (SRZ/ SRZ-P); 38. Vineland Adaptive Behaviour Scales (VABS) |

*Table 5. List of Batteries*

| Batteries |
| --- |
| 1. ACTB - Arizona Cognitive Test Battery 2. Cambridge Cognition Examination (CAMCOG) 3. CAMDEX (Cambridge Mental Disorders of the Elderly Examination) 4. CANTAB - Cambridge Neuropsychological Test Automated Battery 5. Crayton and Oliver Dementia Battery (CaODB) 6. Das-Naglieri Cognitive Assessment System (CAS) 7. ISADYLE language assessment battery (ISADYLE) 8. NAID object memory and memory for sequences 9. Neuropsychological Test series for Elderly with Mild Intellectual Disability (NPEMID) 10. Severe Impairment Battery (SIB) 11. The Later Life Planning Inventory (LLPI) 12. Wechsler Adult Intelligence Scale (WAIS/WAIS-R) 13. WISC-R (Wechsler Intelligence Scale for Children-Revised) 14. WPPSI-R (Wechsler Preschool and Primary Scale of Intelligence-Revised) |

*Table 6. List of Tests*

| Tests |
| --- |
| 1. Autobiographical Memory Test (ABMT) 2. Block design (BD) 3. Block design downward extension (BDDE) 4. BT-ID Barcelona Test-Intellectual Disability 5. Buschke Memory test (BMT) 6. Buschke Verbal Selective Reminding 4-6 years version (BVSR) 7. Conventional Verbal Metaphor Test (CVMT) 8. Corsi block tapping task (CBTT) 9. Corsi Blocks (CB) 10. Developmental Test of Visual Motor Integration (DTVMI) 11. Experimental Computerized Test (ECT) 12. Finger-Nose Test (F-NT) 13. Foundation Aphasia Netherlands Test (FANT) 14. Hiskey-Nebraska Test of Learning Aptitude (HNTLA) 15. Homophone Meaning Generation Test (HMGT) 16. IBR Mental Status Exam (IBR-MSE) 17. Iowa Cognitive Abilities Test (ICAT) 18. KBIT-2 - Kaufman Brief Intelligence Test, Second Edition 19. Matrix Analogies Test-Expanded Form (MAT) 20. Metaphoric Triad Test (MTT) 21. Mini Mental State Examination (MMSE) 22. Modified Cued Recall Test (mCRT) 23. NEPSY comprehension test (NEPSY) 24. Neuropsychological Test series for Elderly with Mild Intellectual Disability (NPEMID) 25. Novel Verbal Metaphor Test (NVMT) 26. Objective Memory Test (OMT) 27. Peabody Picture Vocabulary Test (PPVT) 28. Peabody Picture Vocabulary Test-Revised, Form M – (PPVT-R) 29. Picture Recognition Memory Test (r- PRMT) 30. Purdue Pegboard (PP) 31. Raven Coloured Progressive Matrices (RCPM) 32. Reiss Screen (RS); 33. Rivermead Behavioural Memory Test for Children (RBMT- C) 34. Stanford-Binet ration IQ (S-B IQ) 35. Test for Severe Impairment (TSI) 36. Test of Auditory Comprehension of Language-3 (TACL-III) 37. Test of severe impairment-modified (TSI-M) 38. The Boston naming Test (BNT) 39. The Brief Praxis Test (BPT) 40. The Colour Trails Test (CTT) 41. The controlled Oral Word Association Test (COWAT) 42. The Cued Recall Test (CRT) 43. The Fluid Battery (TFB) 44. The Fuld Object-Memory Evaluation (FOME) 45. The modified Objective Memory Test (OMT) 46. The Neuropsychological Behaviour and Affect Profile (NBAD) 47. The Prudhoe Cognitive Function Test (PCFT) 48. The Selective Reminding Test (SRT) 49. The Standard Progressive Matrices (RSPM) 50. TOLdxtm- Tower of London-Drexel University: 2nd Edition 51. Tower of London (ToL) 52. Trail Making Test (TMT) 53. Visuo-Spatial Test (VST) 54. Weigl Colour-Form Sort Test (WCFST); 55. Wisconsin General Testing Apparatus (WGTA) 56. Wolfenbütteler Dementia Test for Individuals with Intellectual Disabilities (WDTIM) |

*Table 7. List of Tasks*

| *Tasks* |
| --- |
| 1. Acting on request (AoR) 2. Auditory delayed verbal memory (ADVM) 3. Auditory sequential memory (ASM) 4. Block tapping span (BTS) 5. Card sorting task (CST) 6. Cats and Dogs (CaD) 7. Colour Ordering (CO) 8. Complex Span (CS) 9. Concentration ( C ) 10. Delayed match-to-sample (DMTS) 11. Delayed Visual Memory (DVM) 12. Design Span (DS) 13. Digit Span backwards (DSB) 14. Digit Span forwards (DSF) 15. Digital Recall (DR) 16. Experimental Computerized Test (ECT) 17. Expressive Attention (EA) 18. Expressive One-word Picture Vocabulary (EOWPV) 19. Figure Memory (FM) 20. Fragmented Pictures (FP) 21. Gait Assessment (GA) 22. Idiom Comprehension (IC) 23. Matching Numbers (MN) 24. Matching shapes (MS) 25. Matching-to-Sample (MtS) 26. Matrices 27. Memory for objects (MfO) 28. Non-Word Repetition (NWR) 29. Non-Word Span (NWS) 30. Number Finding (NF) 31. Object delayed non-match-to-sample (DNMS) 32. Object discrimination learning (ODL) 33. Object Memory (OM) 34. Object Naming (ON) 35. Objetct pointing span (OPS) 36. Orientation (O) 37. Pattern Recognition (PR) 38. Picture Description (PD) 39. Picture Identification (PI) 40. Picture Naming (PN) 41. Planned Search (PS) 42. Receptive Attention (RA) 43. Reversal learning (RL) 44. Scramble boxes (SB) 45. scrambled boxes (SB) 46. Selective Attention-Expressive (SAE) 47. Semantic Fluency Word Generation Task (SFWGT) 48. Sentence Recall (SR) 49. Shoebox memory task (SbMT) 50. Short Term Memory Task (STMT) 51. Simultaneous Coding Tasks (SCT) 52. Simultaneous Verbal (SV) 53. Spatial delayed non-match-to-position (DNMP) 54. Spatial Recognition (SR) 55. Spatial Reversal (SReversal) 56. Speech Rate (SRate) 57. Successive Coding Tasks (SucCT) 58. Synonyms (S) 59. Temporal Orientation (TO) 60. Verbal Fluency (VF) 61. Visual memory test (VMT) 62. Visual Search (VS) 63. Visual sequential memory (VSM) 64. Visuomotor coordination (VMC) 65. Word Recall (WR) 66. Word Series (WS) 67. Working memory (WM) |
