# Supplementary material for: Screening of Cognitive Changes in Adults with Intellectual Disabilities: A Systematic Review
Source: Brain Sci. 2020 Nov 12;10(11):848. doi: 10.3390/brainsci10110848 (PMC7698112; doi:10.3390/brainsci10110848)
Supplement: Supplementary file 1 [file brainsci-10-00848-s001.zip › brainsci-964520-Sup/longitudinal studies final_CT.docx]

**Table A3.** Longitudinal Studies assessing cognitive changes in mixed groups of ID participants

| Article | Study city/country | Population | Intervention | Comparison | Outcomes | Quality Assessment Score  (0/8) |
| --- | --- | --- | --- | --- | --- | --- |
| Gibson et al (1988) | Canada | N=18 DS; N=18 NDS | PPVT  WISC | Cognitive profiles differences in DS and NDS controls; | Younger DS participants, showed less decline in full-scale scores; Cognitive ability, is more stable over time in DS sample; | 7 |
| Harper & Wadsworth (1990) | USA | N=90 | WAIS-R; ICAT | Comparing individuals in cognitive decline | The declining group with initial lower scores had lower levels of adaptive behaviour, were rated as more depressed and had a higher frequency of problem behaviours. | 6 |
| Devenny et al. 1992 | USA | DS N=28; ID/NDS N=5; MR/NDS N=13 | Tasks: O;  ON; VMC; C  MS  Test: BMT | Between groups | All groups showed comparable improvements in performance tasks from initial testing to second testing on memory; Functional deterioration did not occur among adults with DS. | 7 |
| Thompson (1994) | UK | DS=8 | HSSA; DMR;  RCPM; WAIS-R;  MEAMS; | Neuropsychological assessment tools | All patients score below normal population in RCPM;  Difficult sensitivity in the DMR to distinguished between dementia and depression; | 6 |
| Devenny et al. 1996 | USA | DS/MR N=91,  MR/NDS N=64 | IBR-MSE; SRT  VMT; WISC | Comparing groups | All individuals with possible DAT declined in tasks regarding orientation to time, and object naming. | 7 |
| Visser, Aldenkamp & Huffelen 1997 | Netherlands | DS N=307 | ESDC  SSIMR | Comparing scores between instruments | ESDC it is easy to use, and the symptoms can be assessed quantitatively. | 6 |
| Krinsky-McHale, Devenny & Silverman (2002) | USA | DS/DAT N=14;  DS/NO-DAT N=71 | DSDS  SRT | Changes in memory | Participants with early-stage DAT exhibited significantly greater decline over the 3 year period preceding their diagnosis; Decline in SRT distinguished between groups. | 6 |
| Devenny et al. (2002) | USA | ID/NDS N=66;  DS/no DAT N=75  DS/DAT N=19 | mCRT | Comparing between groups; | Participants with DAT had lower total scores that participants without DAT; Poor performance on the adaptation of CRT was associated with early-stage DAT. | 7 |
| Hassiotis et al. (2003) | UK | DS N=14; ID/NDS N=4 males | RCPM; BPVS-II; CAMCOG; DMR; Mini PASADD; ABS-CR2; | Longitudinal comparision | After 2 years, 38.8 % of participants were diagnosed with dementia. | 7 |
| Kittler, Krinsky-McHale & Devenny 2004 | USA | ID/NDS N=28; ID/DS N=42 | WISC-R | Sex-related changes | Male participants with ID no DS performed better than female participants with ID NDS; Females with DS performed better than males with ID in object assembly and block design; | 6 |
| Zigman et al. (2004) | USA | MR/NDS N=117  MR/DS n=126 | DMR  RS  Part I of AMDAB | Comparing between groups | Equivalent or maybe lower risk for dementia between MR participants and general population; | 7 |
| Ball et al. (2004) | UK | DS N=61 | CAMDEX; CAMCOG; | Longitudinal comparison in dementia diagnosis | with a diagnosis of AD at baseline were at least 6 more times likely to diagnosed with AD at time 2; | 7 |
| Oliver et al. (2005) | UK | DS N=57 | Multidomain | Comparing performances between groups on recall stimulus number | Poor performance and decline in performance on delayed response and conditioned associative learning is associated with dementia in DS adults. | 6 |
| Margallo-Lana et al. (2007) | UK | DS N=92 | The PCFT; The ABS | Longitudinal comparison | Participants with low scores and deterioration on PCFT and ABS later showed dementia; | 6 |
| Nelson et al. (2007) | USA | DS N=34  N=19/34 test 1 year later | WAIS-III; NBAP; DMR; and other tests | Compare validity and reliability of instruments. | NBAP was the strongest predictor of dementia-status. Strong correlation between the pragnosia scale scores and the DMR; | 6 |
| Krinsky-McHale et al. (2008) | USA | DS/DA N=5,  DSno dementia N=25 | Several multidomain | Longitudinal comparison | DS adults who developed DA at early stages showed progressive impairment in selective attention and in ability to selectively attend to stimuli. | 7 |
| Adams & Oliver (2010) | UK | DS N=30 | Several multidomain | Longitudinal comparison | Those with cognitive deterioration show a significant decline on measures of executive function between baseline and 16 months follow up; | 7 |
| Lott et al., (2012) | USA | DS/DAT  No seizure N=29;  Seizure group N=24 | SIB; BPT; DMR  VABS | Comparing performance between groups; | Cognitive decline is more marked in demented individuals with DS who have seizures compared to those who do not. | 6 |
| McCarron et al. (2014) | Ireland, USA | DS N=77 | SIB; DSMSE; DLSQ; DMR | Follow-up | After 20 years, 75 individuals developed dementia at a 20-year follow-up; | 6 |
| Makary et. al., 2014 | Australia | Time 2 n=28;  Mild/moderate ID n=20  Severe/profound ID n=8 | PPVT-4; ABDQ  DBC-A | Follow-up | Adults with DS may experience different ageing patterns for behavioral and emotional problems; | 6 |
| Gutman, Moskovic & Jeret (2016) | USA | DS N=14 | NeuroTrax | Follow-up | No significant changes in scores from point to the next in memory, executive function, verbal, visual spatial and global scores; | 6 |
| Doran et al., (2017) | USA | N=1 | BPT; DMR; SIB  RADD; WAIS III  VABS-II | Follow-up | The prevalence of APP disomy in patients with DS resulting from PT21 appears to be very rare on the basis of only 2 cases, including the present report. | 7 |
| Mccarron et al., (2017) | Ireland | N=77 | DLSQ-NIA; DLD  DSMSE; TSI; | Follow-up | Over 20 years follow-up, 97.4 % developed dementia; | 6 |
| Kuske, Wolff, Govert & Muller (2017) | Germany | baseline sample  n=102 ID; n=22 DS; | WDTIM; DSQIDD | Longitudinal assessment of cognitive decline | WDTIM very suitable for mild to moderate ID but limited for severe ID. | 5 |
| Carr & Collins (2018) | UK | DS N=27 of 50 | LIPS; BPVS; WPPSI; RBMT-C; NAID | changes in cognitive abilities over a 50-year period | Tests of dementia showed falling off in performance even for those without confirmed dementia; | 6 |
| García-Alba et al., (2019) | Spain | DS sample N=41; DS-AD n=13; DS-MCI n=14; DS-Control n=14 | CAMCOG-DS; ADVM; WM; DVM; TO | Performance between groups | DS-AD groups showed significant poorer performance in all tests, especially in verbal and working memory; MCI-DS showed poorer performance than control DS in the CAMCOG and DVM. | 7 |
